# Fraxicon for Optical Applications with Aperture ∼1 mm: Characterisation Study

**DOI:** 10.3390/nano14030287

**Published:** 2024-01-30

**Authors:** Haoran Mu, Daniel Smith, Soon Hock Ng, Vijayakumar Anand, Nguyen Hoai An Le, Raghu Dharmavarapu, Zahra Khajehsaeidimahabadi, Rachael T. Richardson, Patrick Ruther, Paul R. Stoddart, Henrikas Gricius, Tomas Baravykas, Darius Gailevičius, Gediminas Seniutinas, Tomas Katkus, Saulius Juodkazis

**Affiliations:** 1 Optical Sciences Centre, ARC Training Centre in Surface Engineering for Advanced Materials (SEAM), Swinburne University of Technology, Hawthorn, VIC 3122, Australia; haoranmu@swin.edu.au (H.M.); danielsmith@swin.edu.au (D.S.); ale@swin.edu.au (N.H.A.L.); rdharmavarapu@swin.edu.au (R.D.); zkhajehsaeidimahabad@swin.edu.au (Z.K.); pstoddart@swin.edu.au (P.R.S.); gseniutinas@swin.edu.au (G.S.); tkatkus@swin.edu.au (T.K.); sjuodkazis@swin.edu.au (S.J.); 2 Melbourne Centre for Nanofabrication, Australian National Fabrication Facility, Clayton, VIC 3168, Australia; 3 Institute of Physics, University of Tartu, W. Ostwaldi 1, 50411 Tartu, Estonia; 4 Bionics Institute, East Melbourne, VIC 3002, Australia; rrichardson@bionicsinstitute.org; 5 Medical Bionics Department, University of Melbourne, Fitzroy, VIC 3065, Australia; 6 Department of Microsystems Engineering (IMTEK), University of Freiburg, 79110 Freiburg im Breisgau, Germany; ruther@imtek.de; 7 BrainLinks-BrainTools Center, University of Freiburg, 79110 Freiburg im Breisgau, Germany; 8 Laser Research Center, Physics Faculty, Vilnius University, Sauletekio Ave. 10, 10223 Vilnius, Lithuania; henrikas.gricius@outlook.com (H.G.); darius.gailevicius@ff.vu.lt (D.G.); 9 Femtika Ltd., Keramikų Str. 2, 10233 Vilnius, Lithuania; tomas.baravykas@femtika.com; 10 WRH Program International Research Frontiers Initiative (IRFI) Tokyo Institute of Technology, Nagatsuta-cho, Midori-ku, Yokohama 226-8503, Japan

**Keywords:** fraxicon, micro-optics, RGB, SZ2080™ resist, direct laser writing

## Abstract

Emerging applications of optical technologies are driving the development of miniaturised light sources, which in turn require the fabrication of matching micro-optical elements with sub-1 mm cross-sections and high optical quality. This is particularly challenging for spatially constrained biomedical applications where reduced dimensionality is required, such as endoscopy, optogenetics, or optical implants. Planarisation of a lens by the Fresnel lens approach was adapted for a conical lens (axicon) and was made by direct femtosecond 780 nm/100 fs laser writing in the SZ2080™ polymer with a photo-initiator. Optical characterisation of the positive and negative fraxicons is presented. Numerical modelling of fraxicon optical performance under illumination by incoherent and spatially extended light sources is compared with the ideal case of plane-wave illumination. Considering the potential for rapid replication in soft polymers and resists, this approach holds great promise for the most demanding technological applications.

## 1. Introduction

Ultrafast laser-assisted 3D micro-/nano-fabrication (printing) using additive [1,2,3,4,5,6,7], subtractive [8,9,10], and patterning [11,12,13,14,15,16,17,18] modes of material structuring is becoming popular for a wide range of technological tasks and applications [19,20,21,22,23], with a good understanding of the underlying mechanisms of energy deposition and light–matter interactions [24,25,26]. One of the most promising trends is the rapid prototyping and manufacturing of various micro-optical components merging the refractive, diffractive, waveguiding, and polarisation control, or even combined functionalities [27,28,29,30,31]. Another trend is the inscription of waveguides in glasses and crystals [32,33], as well as the formation of optical vortex generators and optical memory structures via form birefringence of self-organised nano-gratings [34]. Fs laser-fabricated optical elements are useful for beam collimation [35], shaping, imaging [36], telecommunications [37], and sensing [38], with an expanding range of functionalities and applications due to the possibility of miniaturisation and efficient fabrication by direct laser writing [39]. The applications of 3D polymerisation were recently reviewed for micro-mechanical applications triggered by different stimuli: light, temperature, and pH [40]. The use of specialised (undisclosed composition) two-photon absorbing photo-resists, hydrogels, and glass composites developed for commercial 3D printers based on fast scanning and high-repetition-rate fs oscillators is a fast-growing application field [41], with a vision of 3D printing applications of computer-designed complex optical elements [42]. A new pathway of 3D formations out of silica has a resolution down to ∼120 nm using photo-polymerisable resist with a 2%wt. The Irgacure 369 photo-initiator and subsequent calcination was demonstrated recently [43]. Similarly, 3D silica structures can be produced from hydrogen silsesquioxane (HSQ) without any photo-initiator by direct write with an fs laser at very different exposure conditions, low repetition rate (∼10 kHz) and long ∼300 fs pulses [44,45], as well as at a high (∼80 MHz) repetition rate and short ∼120 fs pulses [46]. These demonstrations of 3D SiO_2_ structuring down to nanoscale resolutions were recently extended to high-refractive-index ZrO_2_ resists and, moreover, demonstrated at writing speeds approaching 10 m/s using fast polygon and stepping scanners [47]. Another strategy for high-throughput 3D printing is the use of multi-focus arrays [48,49].

High-resolution 3D printing over large areas remains a formidable challenge, especially for optical applications at shorter wavelengths. Maintaining low surface roughness of one-tenth of the wavelength λ/10 or less adds to the challenge. Making flat micro-optical elements for further miniaturisation and compaction of micro-optical solutions is currently trending, but this is even more challenging for the fabrication of optical micro-lenses and functional structures. For controlled phase patterns in diffractive optical elements this is of particular importance and a phase step should be defined over the narrowest lateral width (a step-like height change).

Control of focusing from tight (<10λ) to loose (>10λ) is dependent on the curvature and diameter of the lens *D* and its focal length *f*, which defines the f number $F_{\#}=f/D$, i.e., the numerical aperture $NA\approx 1/2F_{\#}$, and the imaging resolution of the lens. More demanding precision is required for larger micro-optics with a large NA. For flat optical elements, e.g., Fresnel lenses, the 2π phase height (along the light propagation direction) is defined over the height of wavelength ∼λ, which approaches a comparable lateral width for the most off-centre phase rings. This is a challenging 3D laser polymerisation task demanding the most high-resolution laser printing.

High-resolution structures can be made via photo-initiator-free laser writing at high pulse intensity $I_{p}∼\left(1-10\right)\times 10^{12}$ W/cm^2^ or (1–10) TW/cm^2^ [50]. At such high intensities, the photon energy hν≈1.24/(λ[μm]) [eV] approaches the ponderomotive energy (potential) of an electron, i.e., the cycle-averaged quiver energy of a free electron in an electromagnetic field of light: $U_{p}$ [eV] =9.33×(λ ${\left[\mu m]\right)}^{2}\times I_{p}$ [$\left[10^{14}\;W/{\mathrm{cm}}^{2}\right]$]. For λ∼1μm and $I_{p}\approx 10$ TW/cm^2^, the electron quiver energy during one optical cycle reaches $U_{p}\approx 0.93$ eV, which is comparable to the photon energy hν≈1.24 eV. Hence, photo-ionisation of the polymer matrix (∼99 wt.%) can take place without a photo-initiator, which is usually doped at below 1 wt% for the wavelength-specific two-photon absorption. Nonlinear or defect-based absorption provides free electrons which promote further ionisation and chemical-bond breaking via the ponderomotive channel and avalanche ionisation.

Another effective polymerisation pathway is via high-megahertz-repetition-rate exposure of a photo-resist, which facilitates thermal accumulation and cross-linking, even with a very small initial temperature augmentation due to low sub-1 nJ pulse energies. A low thermal diffusivity of the glass substrate and resist $D_{T}=\chi /\left(c_{p}\rho \right)\approx 7\times 10^{-7}$ m^2^/s [51] enhance the local temperature rise (here, χ≈1 W·m^−1^ · K^−1^, and is the thermal conductivity; ρ≈2.2 g/cm^−3^, and is the mass density; and $c_{p}\approx 700$ J/(kg·K), and is the heat capacity at constant pressure). High-repetition-rate laser writing was, therefore, used in this study. Ionisation of the photo-resist modifies the real and imaginary parts of the refractive index $\tilde{n}=n+i\kappa$, i.e., permittivity $\varepsilon \equiv {\tilde{n}}^{2}$, which defines the energy deposition. When the real part of the permittivity $\epsilon_{re}\equiv \left(n^{2}-\kappa^{2}\right)\to 0$ (epsilon-near-zero, ENZ) enters $0<\epsilon_{re}<1$, the most efficient energy deposition into the resist takes place [24]. It is noteworthy that the condition of $\varepsilon_{re}=0$ (or n=κ) defines a runaway process of dielectric breakdown at the focus, which should be avoided for high precision and resolution of 3D polymerised structures.

Designing and manufacturing 3D polymerised structures for beam modulation is more challenging in an integrated optics framework. In most research reports, including distributed Bragg reflector (DBR) laser-based optics, where the periodicity of grating elements is parallel to the beam propagation [52], and cases where the periodicity is perpendicular to the beam propagation [53], binary elements are manufactured. This is because the short integration distance often demands short-period grating elements, and achieving multiple levels within that short period is challenging.

**Figure 1:**
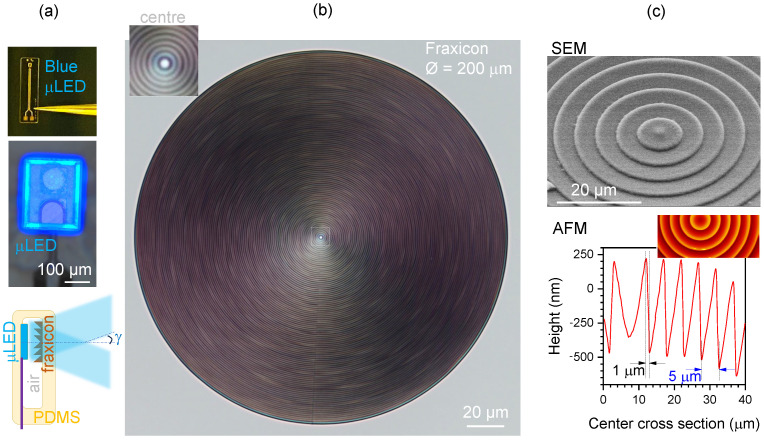
(**a**) Blue micro-LED assembled on a polyimide substrate chip with 460 nm emission and concept design of a flat fraxicon for endoscopy applications made out of silicone (polydimethylsiloxane—PDMS). (**b**) Optical microscope image of a fraxicon made by direct laser writing at 780 nm/100 fs/100 MHz (C-Fiber 780 Erbium Laser, MenloSystems) in SZ2080™ resist. Fraxicon has Λ=1μm period. (**c**) Structural characterisation of fraxicon using SEM and AFM, showing blazed 2π steps with period Λ=5μm.

Here, we demonstrate 3D laser printing of a 0.2 mm diameter fraxicon [54] (flat conical lens) with a triangular phase profile (2π over ∼1 μm height) at 5 μm width in an SZ2080 resist for integration with a micro light-emitting diode (micro-LED; see Figure 1). The lateral step width was defined within ∼1 μm. Characterisation of the fraxicon performance was carried out with optical microscopy and several optical numerical modelling methods: ray tracing, an analytical solution for Gaussian input, the Rayleigh–Sommerfeld (RS) diffraction integral, and holographic simulation.

## 2. Materials and Methods

### 2.1. Resist SZ2080^TM^

The popular hybrid organic–inorganic silica–zirconia composite SZ2080^TM^ (IESL-FORTH, Crete, Greece) was used in this study. Its composition is open (in spite of it being a commercial product) with the identities and concentrations of the photo-initiators being known. This makes the determination of the linear and nonlinear portions of the absorbed energy contributing to the final polymerisation at the defined pulse intensity straightforward, as shown in Ref. [24]. The instantaneous permittivity, square of the complex refractive index, $\varepsilon \left(t\right)\equiv {\left(\tilde{n}\left(t\right)\right)}^{2}$ defines the amount of the absorbed pulse energy as ε(t) changes during the pulse and follows the intensity envelope $I_{p}\left(t\right)$.

SZ2080^TM^ has several properties contributing to its wide use in very different applications: it has a solid (gel) state, high accuracy and resolution of 3D laser printing at the nanoscale, ultra-low shrinking, a small change in refractive index during laser exposure, and a high mechanical stability [55]. It is used for producing nano-photonic structures [55], cell scaffolds [56], biomedical applications [57], micro-optical elements [28,35,38], and functional structures for micro-fluidics [58]. Among other advantages, also useful for this study, are the glass-matching refractive index [55,59], mechanical stability [60], chemical inertness [61], high resilience to laser-induced damage [62], possibility of chemical doping [63], and simple fictionalisation of the fabricated 3D surfaces [64]. Recently, it has been used in thermal post-processing for down-scaling [65] and material-morphing [61]. Interestingly, in addition to all the aforementioned benefits it also allows the possibility of converting the material into an inorganic substance, which enables the realisation of the 3D printing of glass at the nanoscale [61].

### 2.2. Laser Printing Resist

3D laser printing of fraxicons was performed using 780 nm/100 fs (C-Fiber 780 Erbium Laser, MenloSystems, Martinsried, Germany) tightly focused conditions, using an objective lens of numerical aperture NA=1.4 with a beam diameter saturating the input aperture for optimal resolution. The radius at focus was r=0.61λ/NA≈340 nm. The pulse repetition rate was 100 MHz. A combination of fast galvanometric scanners and synchronised precision positioning stages was used, similar to the system described in [66]. The scan velocity varied along the structure and depended on the distance to the geometric centre. The writing strategy was to scan the structure concentrically in closed loops and iterate each next loop by a displacement of the relative radius and height of Δr=50 nm (see discussion below on thermal accumulation) and Δz=300 nm. In the centre, the beam’s travel speed was $v_{sc}=10^{2}\;\mu$m/s and increased linearly to the edge, reaching $10^{3}\;\mu$m/s. Also, kinematic commands, known as rapid jumps (G0), were not used to perform all movements at the same accelerations. This strategy minimises the kinematic fabrication error, where smaller radius loops result in greater excentrical acceleration and scan deviations. For simplicity, we did not account for the cumulative dose variation; therefore, the structure features a slight spherical ramp along the radial direction. Each loop had a ramp-up and ramp-down segment of 30 degrees. A previous study showed that the difference in exposure dose affects the final refractive index and optical performance of a micro-lens, which is better described by wave optics [67]; however, such intricate control of the index by polymerisation was not investigated for the fraxicon fabricated in this current study.

A commercial SZ2080™ resist was used for 3D printing with 2-benzyl-2-dimethylamino-1-(4-morpholinophenyl)-butanone-1 (IRG369, Sigma Aldrich, Darmstadt, Germany) as the photo-initiator dissolved in the initial pre-polymer at 1% wt. The peak of IRG369 absorbance (in SZ2080™) was at 390 nm with emission at 400 nm, as determined by photoluminescence excitation spectrosopy [50]; for pure SZ2080™, absorption and emission were at 350 nm and 400 nm, respectively. To improve the resolution, pure SZ2080™ is preferable; however, a resist with a photo-initiator was used for a larger laser processing window. The energy of a single pulse, with transmission losses accounted for at the focal point, was $E_{p}\approx 96$ pJ, corresponding to fluence per pulse of $F_{p}=0.0275$ J/cm^2^ and intensity of $I_{p}=0.275$ TW/cm^2^ (average). Consequently, the pulses generated a negligible ponderomotive potential. The dwell time required for the beam to cross the focal diameter 2r was $t_{dw}=\frac{2r}{v_{sc}}=6.8$ ms and, at the repetition rate *R*, the number of accumulated pulses over the focal spot was large $N=t_{dw}R=680\times 10^{3}$. The thermal spread (cooling) of the laser-heated focal volume was defined by the time $t_{th}={\left(2r\right)}^{2}/D_{T}=660$ ns, while the time separation between pulses was only 1/R=10 ns. Hence, a very strong thermal accumulation takes place with the used direct laser writing. An average temperature drop at the arrival of the next pulse occurs due to heat transfer to the surrounding cold material. The temperature accumulation $T_{N}$ can be explicitly calculated for the *N* pulses, where a single-pulse temperature jump is $T_{1}$ [68]:

(1) $$T_{N}=T_{1}\left(1+\beta +\beta^{2}+\cdots +\beta^{N}\right)\equiv T_{1}\frac{1-\beta^{N}}{1-\beta },$$

where $\beta =\sqrt{\frac{t_{th}}{t_{th}+1/R}}$ is the constant which defines heat accumulation; and β→1 at high repetition rate R→∞. For the used experimental conditions, β≈0.9925. The first N=10 pulses cause a significant temperature jump $T_{N}=9.67T_{1}$ (N=10). Considering the exothermic character of polymerisation, a minute temperature rise at the focal region causes a guided thermal polymerisation [69].

The samples were prepared by drop-casting the liquid resin on a standard microscope cover slip and pre-condensing at 50 °C for 24 h. After exposure, the samples were developed in methyl-isobutyl-ketone for 30 min, then rinsed with pure developer and air-dried under normal room conditions. The refractive index of the resist at visible wavelengths was approximately $n_{SZ}\approx 1.5$. While the exact definition of the 2π phase height was experimentally challenging for smaller-period fraxicons, the height of the polymerised phase ramps normalised by the wavelength (or 2π in phase) is $h\times n_{SZ}/\lambda ∼2$, which corresponds to the second-order (2×2π) phase steps. This strategy was used for fabrication because of the more straightforward definition of the exact required geometry with a focused laser pulse, which occupies a defined volume.

For more widespread practical implementation of the proposed 3D laser printing of micro-optical elements, the fabrication conditions were optimised to complete the entire laser writing step within 100 min for all 200 μm diameter fraxicons in this study.

### 2.3. Characterisation

Micro-LEDs (CREE C460TR2227, CREE, Durham, USA) were used for the design and prototyping of a low-profile implantable device. The footprint of the μLED was 0.27×0.22 mm^2^, with an emitter area comparable to the D=0.2 mm diameter fraxicon. The micro-LEDs were assembled on a 10 μm thick polyimide substrate [70,71], which was formed by first spin-coating a 5 μm thin polyimide layer on a silicon wafer (diameter 100 mm), followed by the sputter deposition of a metallic thin film. Interconnecting tracks were then patterned using lift-off technology and the track thickness was increased by electroplating 1μm of gold to reduce the electrical line resistance. A second polyimide layer was subsequently deposited to insulate the metal tracks.

**Figure 2:**
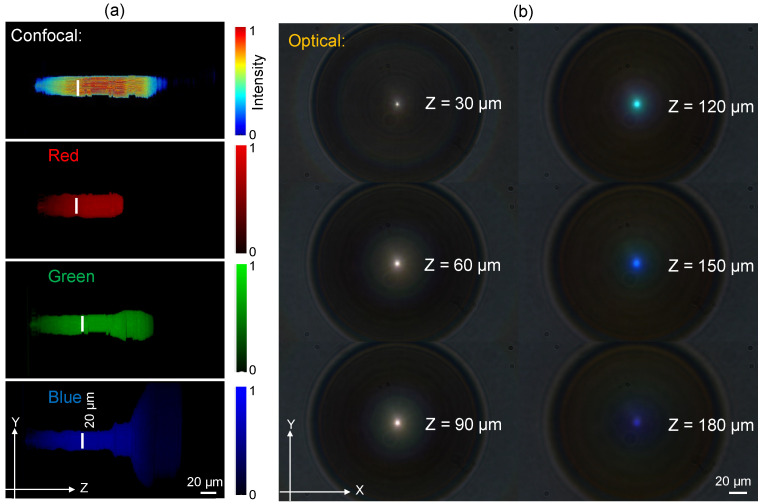
Optical characterisation of fraxicon shown in Figure 1b using an optical microscope with white condenser illumination. (**a**) Confocal intensity distribution and its RGB colour content along the focus (a “non-diffracting” part of the axial intensity). (**b**) Optical images at different axial positions along the white light propagation direction (along *z*-axis).

**Figure 3:**
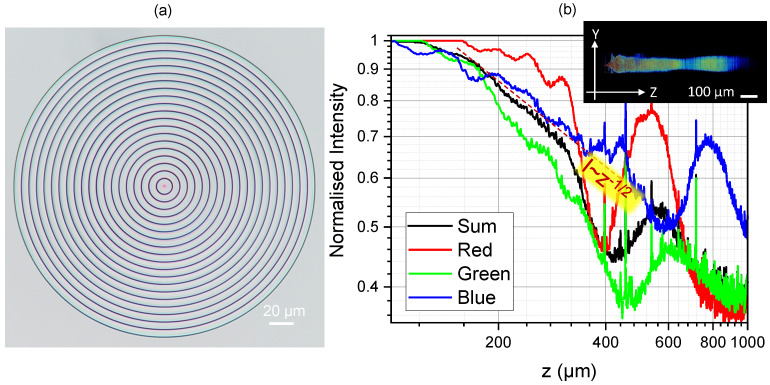
(**a**) Optical image of fraxicon with 1.5-times-larger width of the 2π steps. Image taken under white light condenser illumination. (**b**) Axial intensity profile calculated from the lateral image stacks (same as in Figure 2). The inset shows the confocal profile of intensity.

To access the metal tracks, small openings were formed in the top polyimide layer by reactive-ion etching (RIE) with oxygen plasma. A second metallisation and electroplating step was used to define “bonding pads” for the micro-LED chips and zero-insertion-force (ZIF) connector pads for wire bonding the test structure to a printed circuit board. Finally, the shape of the polyimide substrate was defined by trenching the stack of polyimide layers down to the silicon substrate with a second RIE process step. The substrates could then be peeled from the silicon wafer using tweezers and the micro-LED chips were assembled on the pads of the polyimide substrate by flip-chip bonding [70,71]. They were subsequently underfilled with a biocompatible adhesive (EPO-TEK 301-2, Epoxy Technology, Inc., Billerica, MA, USA) to electrically insulate the pads located at the interface between the micro-LED chips and polyimide substrate. Structural characterisation of the fraxicon was carried out using optical microscopy, scanning electron microscopy (SEM), and atomic force microscopy (AFM). Typical results are shown in Figure 1 for structural and Figure 2 and Figure 3 for optical characterisation, respectively.

### 2.4. Fraxicon: Basic Properties and Design

The positive fraxicon was designed with a diameter of D=0.2 mm and featured 20 blazed rings. The thickness profile ($t_{fra}$) of the fraxicon as a function of the radial coordinate ($r=\sqrt{x^{2}+y^{2}}$) is given as shown in Equation (2):

(2) $$t_{fra}=h-mod\left[r\times \left(\frac{h}{\Lambda }\right),h\right]$$

where *h* is the height of the fraxicon corresponding to 2π phase retardation ($h=\frac{\lambda }{\left(n_{SZ}-1\right)}=1\;\mu$m, λ is the incident wavelength, $n_{SZ}\approx 1.5$ is the refractive index of SZ2080™), Λ=5μm is the period of the gratings (rings), and mod is a function of the remainder after division (modulo operation).

The axial intensity AI(z) distribution (along the z-axis) of an axicon/fraxicon depends on the radial intensity at the input and can be found from a relation based on Snell’s law r=z(n−1)α; here, *n* is the refractive index of the axicon and α is the base angle of the axicon (also, the angle required to the full π angle at the tip of the axicon). For the Gaussian beam $I_{in}=I_{0}\exp\left(-\frac{2r^{2}}{w_{o}^{2}}\right)$, where $w_{o}$ is the beam waist,

(3) $$AI\left(z\right)=I_{0}\exp\left(-\frac{2z^{2}{\left(n-1\right)}^{2}\alpha^{2}}{w_{o}^{2}}\right)\times \left[2\pi z{\left(n-1\right)}^{2}\alpha^{2}\right].$$

This equation can be generalised and the input intensity profile $I_{in}\left(r\right)$ can be expressed in terms of *r*-to-*z* mapping AI(z) as $I_{in}\left(r\right)=M\times AI\left[z=r/\left(n-1\right)\alpha \right]/r$, where $M=\frac{1}{2\pi (n-1)\alpha }$ is the axicon geometry-defined constant. This is valid for the on-axis intensity in the 0<z<DOF region with the depth of focus DOF=w/[(n−1)α] defined by the radius of beam *w*.

## 3. Results

### 3.1. Theory: Fraxicon Illuminated by an Incoherent and Extended Source

**Figure 4:**
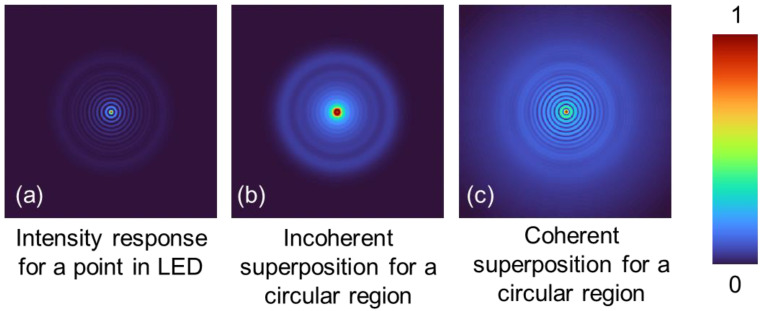
(**a**) Intensity distribution obtained for a single point of the LED. (**b**) Intensity distribution obtained for a circular region of the LED by incoherent superposition. (**c**) Intensity distribution obtained for the same circular region as (**b**) but with coherent superposition.

Gauss–Bessel beam generation by fraxicons is mostly discussed using spatially coherent illumination such as laser beams. The timing of photon emission from the LED is, however, disorganised. To describe beam generation by fraxicons for a spatially incoherent illumination, such as light from an LED, an incoherent imaging framework is needed. The LED needs to be considered as a collection of points and the generated beam is formed by the summation of intensities of the beam generated for every point. While it is difficult to discriminate beams generated for coherent and incoherent illuminations, the generation approaches are quite different from one another. We consider a point in the LED as a Delta function emitting a spherical wavefront with intensity $\sqrt{I_{s}}$, given as $S\left(1/z_{s}\right)=\exp\left[j\left(2\pi /\lambda \right)\sqrt{x^{2}+y^{2}+z_{s}^{2}}\right]$. The phase of the fraxicon is given as $\phi =\exp[j\left(2\pi /\lambda \right)t_{fra}]$. The complex amplitude after the fraxicon is, therefore, $\psi =\sqrt{I_{s}}C_{1}L\left(r_{s}/z_{s}\right)S\left(1/z_{s}\right)\phi$, where *L* is a linear phase and $C_{1}$ is a complex constant. The intensity distribution at a distance $z_{r}$ for a Delta-function is given as $I_{Delta}\approx {\left|\psi ⨂Q\left(1/z_{r}\right)\right|}^{2}$, where $Q\left(1/z_{r}\right)=\exp\left[j\left(\pi /\lambda z_{r}\right)\left(x^{2}+y^{2}\right)\right]$ and ‘⨂’ is a 2D convolutional operator. The intensity distribution obtained for the entirety of the LED’s active area can be given as $I_{LED}\approx {\left|I_{Delta}⨂O\right|}^{2}$, where *O* is the LED’s active area in a square shape filled with ones and zeros around it. It must be noted that the above summation is not a complex summation but an addition of intensities, as the phase information is not present. The above expression is an approximate one as Fresnel approximation was used for propagation between the fraxicon and the camera. To understand the beam generation more deeply, let us consider the Delta function with no linear phase attached to it, i.e., the one at the centre of the LED’s active area. This Delta function generates a spherical wave that interacts with the fraxicon. We already established that fraxicons and axicons consist of lens functions with different focal lengths multiplexed in the transverse direction [72]. At the camera plane, one of the lens functions satisfies the imaging condition, generating a sharp Delta-like function. The other lens functions cause ring patterns around this sharp Delta-like function, typical of a squared Bessel function. The other points in the LED’s active area with linear phases attached to them create off-axis squared Bessel functions on the camera. The recorded intensity distribution is the sum of all the contributions from the points of the LED’s active area. This is different from coherent illumination as it would depict the behaviour of light emission from a single point. Simulation results for the intensity obtained for a point in an LED, the intensity obtained for a circular region in an LED by incoherent superposition due to spatial incoherence, and the intensity obtained for the same circular region but with coherent superposition are shown in Figure 4a–c, respectively. The incoherent superposition does not generate distinct rings around the central maxima as expected of a Bessel distribution due to the lack of phase relations for light emitted from every point. The temporal coherence length for a Gaussian fit of LED emission spectra is given by $L_{tc}=\frac{4\ln2}{\pi }\frac{\lambda_{0}^{2}}{\Delta \lambda }$, where $\lambda_{0}$ is the central emission wavelength and Δλ is its width or full width at half maximum (FWHM), and it coincides well with experimentally measured values $L_{tc}\approx 2\;\mu$m using Mach–Zehnder interferometry [73].

### 3.2. Experimental Characterisation of Light Intensity

An axicon or a conical lens is a very useful optical element that forms an elongated axial intensity distribution when illuminated by a Gaussian beam. The formed Bessel–Gaussian beam has a diameter defined by the first minimum of the Bessel function $J_{0}\left(k_{⊥}r\right)$, where *r* is the radial coordinate in the lateral plane, and the perpendicular component of the propagation wavevector $k_{⊥}$ is defined by $k_{⊥}\equiv k\sin\gamma$, with γ being the half-cone angle of the beam with an optical axis, wavevector k=ω/c≡2π/λ, and $k_{⊥}^{2}+k_{\|}^{2}={\left(\omega /c\right)}^{2}$; ω and *c* are the cyclic frequency and speed of light, respectively. The diameter of the axially extended focus is $d_{B}=4.816/k_{⊥}$ and the length depends on the diameter of the incident beam *D* (lens diameter) as $Z_{max}=D/\left(2\sin\gamma \right)$. A large *D* and small γ facilitates having a long, so-called, non-diffracting region of intensity on the optical axis. An axicon can be made flat in the same way as a Fresnel lens is made from concentric segments.

A fraxicon lens of D=300μm, consisting of 30 circular rings of ∼5 μm width and 0.7μm height, was polymerised in SZ2080™ using fs laser direct write (Figure 1). The step between adjacent phase ramps was within ∼1 μm. Fraxicons are especially promising for optical devices which have strong requirements for spatial constraints. If fraxicons are used for optical focusing of micro-light sources, e.g., LEDs, it is usual that illumination of the aperture of the fraxicon will take place from an extended, non-collimated light source with possible intensity inhomogeneities on a scale of tens of micrometers (see powered μLED image in Figure 1a). Such a situation can be modelled using an optical microscope under condenser illumination of a fraxicon on a sample plane, as discussed next.

#### Confocal Intensity Mapping

Figure 2a shows the axial intensity distribution calculated from the axial stacks, as shown in Figure 2b, using a standard microscope (Nikon, Tokyo, Japan, Optiphot) under white light condenser illumination in transmission mode. Images at every Δz=1μm step were recorded and the 3D intensity distribution was calculated using our own Matlab code. The resulting confocal axial intensity distribution was separated into basic red, green, blue (RGB) colour channels. The width of the focal region was $d_{B}\approx 20\;\mu$m; hence, $k\sin\gamma =4.816/d_{B}$ or $\gamma \approx 1.1^{\circ }$ for λ≈0.5μm. As expected, the width for R-red axial distribution $d_{B}^{\left(R\right)}$ was larger than for B-blue, $d_{B}^{\left(B\right)}$. Strong widening of the intensity profile for the blue channel is most probably defined by the comparatively high NA of 0.9 of the imaging microscope lens, since the conical angle $\gamma \approx 1.1^{\circ }$ is small.

The length of the most uniform intensity region is wavelength-dependent since the height of the phase ramps is fixed. The longest non-diffracting region was for the blue wavelength. This is also clearly seen in the lateral cross-sections at z>150μm (Figure 2b).

Figure 3 shows the fraxicon and its axial intensity cross-sections under condenser illumination in the microscope. The period of concentric phase ramps was 1.5 times larger, hence ∼7.5 μm, while the diameter and the height of the phase ramps were the same as for the fraxicon in Figure 1b and Figure 2. This resulted in a longer non-diffracting region. The intensity decreased along the propagation direction as $\sqrt{z}$ and the RGB colours had different effective lengths. The exact axial intensity profile depends on the wavevector spread for different colours in the illumination light. The ideal performance of fraxicons was modelled numerically and is discussed next.

### 3.3. Numerical Predictions from Ray and Wave Simulations

For numerical simulations we used the ideal case of triangular phase steps of 20 rings, with each of them having 5 μm width and 1μm height. The material of the phase steps had a refractive index of 1.5 (dispersion free). This made a D=0.2 mm diameter fraxicon, which was illuminated with a plane wave (a singe wavenumber) at different RGB colours.

#### 3.3.1. Wave Optics

**Figure 5:**
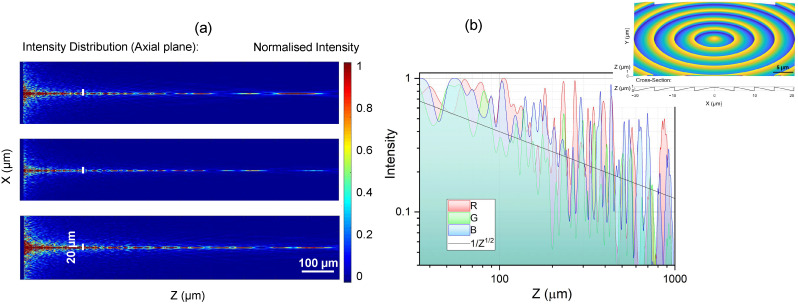
(**a**) Simulation using the Rayleigh–Sommerfeld (RS) diffraction integral for non-polarised plane wave with RGB wavelengths: R = 700 nm, G = 546.1 nm, and B = 435.8 nm (top-down). The calculated intensity cross-section is given along the propagation direction. (**b**) Central intensity cross-section for the RGB colours; inset shows geometry of simulated positive fraxicon with Λ=5μm period.

The Rayleigh–Sommerfeld (RS) diffraction integral can be used for exact prediction of the axial intensity profile, which is consistent with the wave-optical approach; it is used for thin graphene micro-lenses [74]. The electric field distributions $U_{2}\left(r_{2},z\right)$ in the axial plane at different axial *z* positions were calculated using the MATLAB program based on the RS diffraction integral expressed as

(4) $$U_{2}\left(r_{2},\theta_{2},z\right)=\frac{-i}{\lambda }\;\int \int \;U_{1}^{\prime }\left(r_{1},\theta_{1}\right)\cdot \frac{e^{-ikr}}{r}\cdot \cos\left(n,r\right)dr_{1}d\theta_{1},$$

where λ is the incident light wavelength (R = 700 nm, G = 546.1 nm, and B = 435.8 nm), $k=\frac{2\pi }{\lambda }$ is the wave vector, ($r_{1},\theta_{1}$) and ($r_{2},\theta_{2}$) are the polar coordinates in the diffraction plane (the plane immediately behind the fraxicon) and observation plane (the focal plane), respectively; $r=\sqrt{(z^{2}+{\left(x_{2}-x_{1}\right)}^{2}+{\left(y_{2}-y_{1}\right)}^{2}}=\sqrt{(z^{2}+r_{1}^{2}+r_{2}^{2}-2r_{1}r_{2}cos\left(\theta_{1}-\theta_{2}\right)}$, cos(n,r) is defined as the cosine of the angle between the unit normal vector n of the diffraction plane and the position vector r from point $\left(r_{1},\theta_{1}\right)$ to point $\left(r_{2},\theta_{2}\right)$, and $U_{1}^{\prime }\left(r_{1},\theta_{1}\right)$ is the E-field immediately behind the fraxicon. The incident wave $U_{1}\left(r_{1},\theta_{1}\right)$ is diffracted by the fraxicon through amplitude and phase modulations, and the electric field modified by the fraxicon $U_{1}^{\prime }\left(r_{1},\theta_{1}\right)$ can be expressed by Equation (5):

(5) $$U_{1}^{\prime }\left(r_{1}\right)=U_{1}\left(r_{1}\right)\cdot \sqrt{T(r_{1})}\cdot e^{-ik\cdot \Phi (r_{1})},$$

where $T(r_{1})$ is the transmission distribution (amplitude modulation) of the fraxicon, and $\Phi \left(r_{1}\right)=n_{SZ}\cdot t_{fra}$ ($n_{SZ}$ is the refractive index of SZ2080™, and $t_{fra}$ is the thickness profile of the fraxicon) is the phase modulation provided by the fraxicon. Consequently, the light intensity distributions in the axial plane can be calculated by squaring the electric field: $I\left(r_{2},z\right)={\left|U_{2}\left(r_{2},z\right)\right|}^{2}$ (Figure 5).

Apparent differences between the modeling of ideal plane-wave illumination of a fraxicon (Figure 5) and experimental imaging using condenser illumination of the microscope (Figure 3) is due to the presence of different *k* components at the same wavelength. Such a situation is expected in real applications where the fraxicon is placed in front of a μLED (Figure 1a). However, the basic features of the oscillatory nature of intensity along the propagation axis, its decay as $\propto \sqrt{z}$, and sub-1 mm long extent of the high-intensity section are consistent. Exact intensity distributions can be well controlled using tailored illumination of an axicon [75], and hence, a fraxicon as well.

#### 3.3.2. Ray Optics

**Figure 6:**
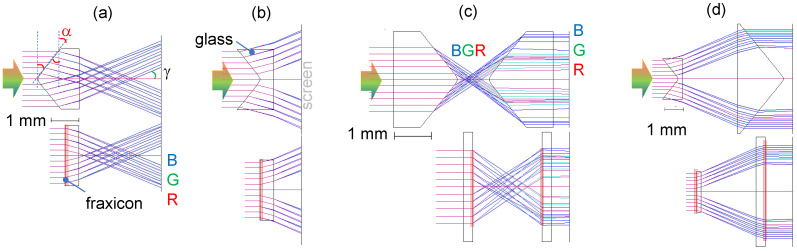
Ray tracing through (fr)axicons and their pairs (OSLO, Lambda Research Co.). Positive (**a**) and negative (**b**) fraxicon and axicon. Pairs of (fr)axicons (i.e., collimating telescopes): two positive (**c**) and a negative–positive pair (**d**) for the bulk- and flat-optics realisations. Illumination with RGB colour light shown by arrow. Location of the planarised (fraxicon) region is shown by shaded markers. The base angle of the axicon is α and γ is the half-cone angle of the Bessel beam. The refractive index of glass was n=1.52 and the base angle $\alpha =\pm 40^{\circ }$, where the positive sign is for real focus (**a**) and negative for virtual focus (**b**); a large angle was chosen for visualisation and a strong angular dispersion of RGB rays.

The Optical Software (Version 6.3) for Layout and Optimisation (OSLO, Lambda Research Co., Westford, MA, USA) is a ray-tracing tool used to model light propagation through (fr)axicons. Figure 6 shows RGB beam propagation through positive and negative (fr)axicons and their pairs, which collimates the beam. Change from an axicon to a fraxicon is conveniently made by tab-selection in the OSLO input; the fraxicon surface appears flat in the viewer; however, it encodes a fraxicon layout of 2π phase ramps. As expected from the shape of the axicon, a conical prism, light dispersion is evident in the RGB ray tracing (Figure 6). It manifests as a colour aberration along the focal region. A slight colour appearance of the fraxicon when imaged with a microscope is evident in Figure 3a, with a red centre and blue outside the edges of the phase ramps. Figure 6 clearly shows the compactness of micro-optical constructions using fraxicons and less dispersion in a pair of the flat optical elements Figure 6c,d for the same diameter of input beam; the alignment in Figure 6 is at the plane of the screen.

Such compact fraxicons have the potential for application in space telescope technologies. It is planned to use multiple-order diffractive-engineered surface (MODE) lenses, which comprise a front-surface multiple-order diffractive lens (MOD) and a rear-surface diffractive Fresnel lens (DFL), to aid in the search for Earth-like planets and exoplanets in the universe as part of the upcoming telescope array known as the Nautilus Observatory [76,77,78].

## 4. Discussion

The circular grating pattern makes an efficient collection of illumination at different wavelengths onto the optical axis and is useful for light delivery and collection to spot sizes of tens of micrometres. The long focal extension is helpful for light coupling into optical fibres and laser machining using increasingly miniaturised laser sources. Photonic crystal (PhC) lasers now deliver output powers of tens of watts at near-IR wavelengths from sub-1 mm apertures at a very low divergence angle [79]. Micro-optics based on a high-damage-threshold SZ2080™ photo-resist is a promising solution [80].

Gauss–Bessel beams formed by phase profiles closely matching those of a fraxicon were made with a spatial light modulator for record high ∼10^4^ aspect-ratio modification of dielectrics and semiconductors for scribing and dicing with a nanoscale resolution of tens of nanometres [81]. For such material modification, multiple ionisation locations along the non-diffracting intensity distribution are essential and their connection occurs via back-scattering under multi-pulse irradiation. Hence, the oscillatory nature of the Bessel beam intensity on the axis (Figure 5) is beneficial for such material modifications.

The replication of polymerised fraxicons can be achieved using Ni-shim (plasma coating with subsequent electrochemical deposition of Ni), which replicates structures with 10 nm feature sizes, e.g., nano-needles of black Si [82].

The emergence of a new generation of optical cochlear implants has highlighted the need for better miniaturisation and integration of optical elements. It has been shown that optical neuromodulation with visible light, facilitated by optogenetics, can confer higher spatial precision of neural activation compared to traditional electrical stimulation methods [83]. For cochlear implants, a broad current spread from electrical devices reduces the number of independent stimulating channels [84,85]. A higher spatial precision of activation could greatly increase the number of independent stimulating channels and enable simultaneous channel stimulation, which would greatly enrich the sound quality experienced by cochlear implant recipients [86,87]. Strategies to deliver focused electrical stimulation, including tripolar and focused multipolar stimulation strategies, have failed to deliver significant clinical benefit [88,89]. The emerging development of optical arrays could provide an alternative solution.

In rodents, such as mice, rats, and gerbils, auditory neurons were modified to express photosensitive ion channels. The spread of activation during optical stimulation was significantly lower compared to electrical stimulation, resulting in near-physiological spectral resolution when using light emitters that were in close proximity to the neural tissue [71,90,91]. Furthermore, during two-channel simultaneous optical stimulation in the mouse cochlea using micro-LEDs with a pitch of just 0.52 mm, channel interaction was 13–15-fold lower than simultaneous electrical channel stimulation [92]. In the human cochlea, where there is a greater distance between the emitter and the neural tissue, optical arrays of micro-LEDs are still predicted to significantly reduce the spread of activation in the cochlea to 0.4–1.0 octaves, up to fourfold lower than electrical stimulation [93,94]. While modelling data suggests waveguides could provide even greater spectral selectivity [95], the fraxicon technology presented here could potentially be used to focus the emission cone and improve the spectral resolution provided by LEDs in the human cochlea.

An antireflection coating could be used to increase the transmittance of a fraxicon by coating a film with a thickness of λ/4 of a refractive index $\sqrt{n_{out}n_{frax}}\approx 1.25$, where $n_{out}=1$ (air) is the refractive index at the focal region and $n_{frax}=1.5$. MgF_2_ is a good candidate for the antireflection coating over the visible spectral range. Atomic layer deposition (ALD) is a candidate for conformal coating of 3D surfaces, as is magnetron sputtering.

**Figure A1:**
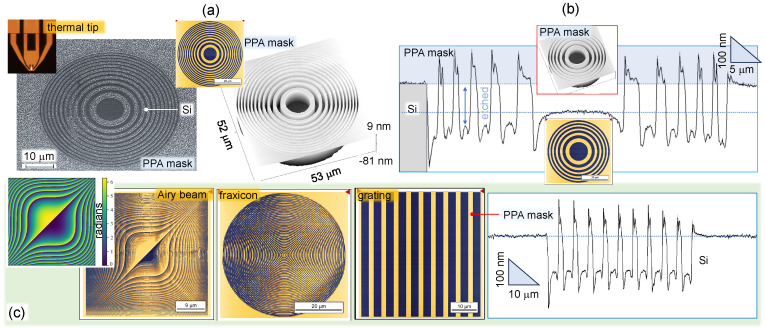
Sacrificial mask etch of micro-optical elements into Si. (**a**) A tilted-view SEM image of a binary Fresnel lens (f=50μm) after RIE plasma etching, optical image (yellow middle-inset), and AFM image of the pattern defined in a PPA resist using thermal tip nanolithography (NanoFrazor; top-left inset shows the writing tip). (**b**) Optical profilometer cross-section of an f=100μm lens; insets show optical and AFM images. (**c**) Microscope images of PPA masks of the cubic-phase structure (Airy beam generator; a phase 0–2π phase map in the inset); four-level fraxicon; binary grating with period of 5 μm. Optical profilometer cross-section of the grating after RIE.

Fraxicons with ∼1μm tall phase ramps can be made using a scanning thermal tip (nano-cantilever) method based on the AFM principle (NanoFrazor; Appendix A). By using a polyphthalaldehyde (PPA) 4% resist for a grey-scale mask, e.g., 3000 rpm spin coating for a 100 nm film, a sacrificial PPA mask can be made with high nanoscale control and resolution down to 10 nm. Such a mask is used for the transfer of the 3D pattern onto a substrate by reactive-ion etching (RIE). For Si etch (Figure A1), the etching contrast was ∼2.6 and translates to 260 nm deep structures when a PPA resist is ∼100 nm. Different patterns and metasurface structures can be easily designed for patterning into the resist using the open-source toolbox [96]. This is based on the industry standard GDSII, a binary database file format for electronic design automation data exchange of integrated circuit layouts.

## 5. Conclusions and Outlook

The 3D printing of 0.2 mm diameter flat fraxicon lenses with 2π phase step defined over 1 and 5 μm lateral widths and ∼0.7 μm height (axial length) was performed in an SZ2080™ (with 1%wt. IRG) resist. Then, 3D polymerisation was carried out using tightly focused 780 nm/100 fs/100 MHz laser irradiation. The high repetition rate and strong overlap of the laser pulses at the focal diameter of 680 nm, with a linear scan step of 50 nm between the adjacent pulses, determined a strong thermal accumulation of 3D laser printing/polymerisation with only ∼0.1 nJ pulses. The used tight focusing, with a depth of focus approximately 3–4 times longer than the focal diameter, i.e., 2.5–3 μm, was significantly shorter than the axial pulse length of $ct_{p}\approx 30\;\mu$m.

The entire fraxicon was printed within 1.5 h. It was tested for illumination using an extended light source (condenser of a microscope) to simulate its performance for μLED illumination in endoscopy and opto-probes. The RGB colour analysis revealed an axial colour separation along the light propagation direction, which is significant for an extended white light source. Different axial intensity distributions are predicted from the analysis of a fraxicon illuminated by incoherent and coherent light sources. Fraxicons with wider sections of phase ramps have fewer diffraction-related effects as compared with flat Fresnel lenses, which have increasingly narrow phase ramps at larger diameters, and consequently, a stronger diffraction. These aspects of fraxicon use in micro-optical applications have to be considered. 

## Data Availability

Data can be made available upon a reasonable request.

## Appendix A. Thermal Scanning Probe Lithography (tSPL)

Scanning thermal tip nanolithography (NanoFrazor, SwissLitho) has a 10 nm resolution (defined by the tip) and the possibility to sublimate/evaporate a solid linear polyphthalaldehyde (PPA; anionic from Sigma-Aldrich) resist upon fast and localised heating. The height control of resist removal is adjustable; hence, a 3D surface can be defined for a sacrificial mask in RIE-ICP plasma etch.

The etch depths were measured using an optical profiler to estimate the etch depth into Si and etch contrast with PPA. Figure A1 shows several examples of a grating, Fresnel lens, and 3D phase patterns of the cubic phase (Airy beam generator) and a four-level fraxicon. The etch depth was approximately around 160–180 nm for a 65 nm deep pattern defined in PPA; the PPA thickness was 100 nm for a 5% solution of PPA spin-coated at 2000 rpm. This translated to an etch selectivity (Si vs. PPA) of 2.6, which is less than the best-performing examples with NanoFrazor, which reach 4–10 depending on the pattern and etch conditions. The most probable reason for the lower selectivity is the introduction of O_2_ into the process, which might have suppressed the selectivity of SF_6_ for Si. The RIE conditions were (Oxford, Plasmalab 100 ICP380) SF_6_ 40 sccm, C_4_F_8_ 60 sccm, O_2_ 10 sccm, at an RF of 40 W, ICP at 1500 W, etch time of 50 s, and pressure of 10 mTorr. The RF strike power was increased to 50 W for the 5 s plasma ignition step only. For binary patterns, a metal hard mask (e.g., Cr) could be used for Si etching via the Bosch process up to a large 150 μm depth, as shown for THz optical vortex generator elements based on a metasurface design [97].

The typical pattern-writing time for 60 nm structures of Fresnel lenses in PPA (Figure A1a) was 5 min for a 50×50μm^2^ area with an f=50μm lens and four-level fraxicon. The residuals of PPA were removed beforehand with gentle barrel washing ∼5 s, 200 W, 400 sccm O_2_. Also, a developer of tetramethylammonium hydroxide (TMAH) can be used.

## References

[1] Maruo S., Nakamura O., Kawata S. (1997). Three-dimensional microfabrication with two-photon-absorbed photopolymerization. Opt. Lett..

[2] Kawata S., Sun H.B., Tanaka T., Takada K. (2001). Finer features for functional microdevices. Nature.

[3] Serbin J., Egbert A., Ostendorf A., Chichkov B.N., Houbertz R., Domann G., Schulz J., Cronauer C., Fröhlich L., Popall M. (2003). Femtosecond laser-induced two-photon polymerization of inorganic–organic hybrid materials for applications in photonics. Opt. Lett..

[4] Sun H.B., Kawakami T., Xu Y., Ye J.Y., Matuso S., Misawa H., Miwa M., Kaneko R. (2000). Real three-dimensional microstructures fabricated by photopolymerization of resins through two-photon absorption. Opt. Lett..

[5] Takada K., Sun H.B., Kawata S. (2005). Improved spatial resolution and surface roughness in photopolymerization-based laser nanowriting. Appl. Phys. Lett..

[6] Sun H.B., Tanaka T., Kawata S. (2002). Three-dimensional focal spots related to two-photon excitation. Appl. Phys. Lett..

[7] Farsari M., Chichkov B. (2009). Two-photon fabrication. Nat. Photonics.

[8] Joglekar A.P., hua Liu H., Meyhöfer E., Mourou G., Hunt A.J. (2004). Optics at critical intensity: Applications to nanomorphing. Proc. Natl. Acad. Sci. USA.

[9] Mao B., Siddaiah A., Liao Y., Menezes P.L. (2020). Laser surface texturing and related techniques for enhancing tribological performance of engineering materials: A review. J. Manuf. Process..

[10] Korte F., Serbin J., Koch J., Egbert A., Fallnich C., Ostendorf A., Chichkov B. (2003). Towards nanostructuring with femtosecond laser pulses. Appl. Phys. A.

[11] Davis K.M., Miura K., Sugimoto N., Hirao K. (1996). Writing waveguides in glass with a femtosecond laser. Opt. Lett..

[12] Miura K., Qiu J., Inouye H., Mitsuyu T., Hirao K. (1997). Photowritten optical waveguides in various glasses with ultrashort pulse laser. Appl. Phys. Lett..

[13] Shimotsuma Y., Kazansky P.G., Qiu J., Hirao K. (2003). Self-Organized Nanogratings in Glass Irradiated by Ultrashort Light Pulses. Phys. Rev. Lett..

[14] Beresna M., Gecevičius M., Kazansky P.G., Gertus T. (2011). Radially polarized optical vortex converter created by femtosecond laser nanostructuring of glass. Appl. Phys. Lett..

[15] Bellouard Y., Said A., Dugan M., Bado P. (2004). Fabrication of high-aspect ratio, micro-fluidic channels and tunnels using femtosecond laser pulses and chemical etching. Opt. Express.

[16] Vitek D.N., Block E., Bellouard Y., Adams D.E., Backus S., Kleinfeld D., Durfee C.G., Squier J.A. (2010). Spatio-temporally focused femtosecond laser pulses for nonreciprocal writing in optically transparent materials. Opt. Express.

[17] Bellouard Y., Said A.A., Bado P. (2005). Integrating optics and micro-mechanics in a single substrate: A step toward monolithic integration in fused silica. Opt. Express.

[18] Liao Y., Ni J., Qiao L., Huang M., Bellouard Y., Sugioka K., Cheng Y. (2015). High-fidelity visualization of formation of volume nanogratings in porous glass by femtosecond laser irradiation. Optica.

[19] Bellouard Y., Champion A., Lenssen B., Matteucci M., Schaap A., Beresna M., Corbari C., Gecevičius M., Kazansky P., Chappuis O. (2012). Integrating optics and micro-mechanics in a single substrate: A step toward monolithic integration in fused silica. J. Laser Micro/Nanoeng..

[20] Osellame R., Hoekstra H., Cerullo G., Pollnau M. (2011). Femtosecond laser microstructuring: An enabling tool for optofluidic lab-on-chips. Laser Photon. Rev..

[21] Sugioka K., Cheng Y. (2014). Ultrafast lasers-reliable tools for advanced materials processing. Light Sci. Appl..

[22] Öktem B., Pavlov I., Ilday S., Kalaycioglu H., Rybak A., Yavas S., Erdogan M., Ilday F. (2013). Nonlinear laser lithography for indefinitely large-area nanostructuring with femtosecond pulses. Nat. Photonics.

[23] Liu H., Lin W., Hong M. (2021). Hybrid laser precision engineering of transparent hard materials: Challenges, solutions and applications. Light Sci. Appl..

[24] Wang H., Zhang W., Ladika D., Yu H., Gailevičius D., Wang H., Pan C.F., Suseela Nair P., Ke Y., Mori T. (2023). Two-Photon Polymerization Lithography for Optics and Photonics: Fundamentals, Materials, Technologies, and Applications. Adv. Func. Mat..

[25] Gamaly E.G., Rode A.V., Luther-Davies B., Tikhonchuk V.T. (2002). Ablation of solids by femtosecond lasers: Ablation mechanism and ablation thresholds for metals and dielectrics. Phys. Plasmas.

[26] Momma C., Chichkov B.N., Nolte S., von Alvensleben F., Tünnermann A., Welling H., Wellegehausen B. (1996). Short-pulse laser ablation of solid targets. Opt. Commun..

[27] Moughames J., Porte X., Thiel M., Ulliac G., Larger L., Jacquot M., Kadic M., Brunner D. (2020). Three-dimensional waveguide interconnects for scalable integration of photonic neural networks. Optica.

[28] Malinauskas M., Zukauskas A., Belazaras K., Tikuisis K., Purlys V., Gadonas R., Piskarskas A. (2012). Laser fabrication of various polymer micro-optical components. Eur. Phys. J.-Appl. Phys..

[29] Schumann M., Bückmann T., Gruhler N., Wegener M., Pernice W. (2014). Hybrid 2D–3D optical devices for integrated optics by direct laser writing. Light Sci. Appl..

[30] Bertoncini A., Liberale C. (2018). Polarization Micro-Optics: Circular Polarization From a Fresnel Rhomb 3D Printed on an Optical Fiber. Photon. Technol. Lett..

[31] Thiele S., Pruss C., Herkommer A., Giessen H. (2019). 3D printed stacked diffractive microlenses. Opt. Express.

[32] Jia Y., Wang S., Chen F. (2020). Femtosecond laser direct writing of flexibly configured waveguide geometries in optical crystals: Fabrication and application. Opto-Electron. Adv..

[33] Li L., Kong W., Chen F. (2022). Femtosecond laser-inscribed optical waveguides in dielectric crystals: A concise review and recent advances. Adv. Photonics.

[34] Sakakura M., Lei Y., Wang L., Yu Y., Kazansky P. (2020). Ultralow-loss geometric phase and polarization shaping by ultrafast laser writing in silica glass. Light Sci. Appl..

[35] Zukauskas A., Malinauskas M., Brasselet E. (2013). Monolithic generators of pseudo-nondiffracting optical vortex beams at the microscale. Appl. Phys. Lett..

[36] Gissibl T., Thiele S., Herkommer A., Giessen H. (2016). Sub-micrometre accurate free-form optics by three-dimensional printing on single-mode fibres. Nat. Commun..

[37] Dietrich P.I., Blaicher M., Reuter I., Billah M., Hoose T., Hofmann A., Caer C., Dangel R., Offrein B., Troppenz U. (2018). In situ 3D nanoprinting of free-form coupling elements for hybrid photonic integration. Nat. Photonics.

[38] Melissinaki V., Farsari M., Pissadakis S. (2015). A Fiber-Endface, Fabry–Perot Vapor Microsensor Fabricated by Multiphoton Polymerization. IEEE J. Select. Top. Quant. Electron..

[39] Gonzalez-Hernandez D., Varapnickas S., Bertoncini A., Liberale C., Malinauskas M. (2023). Micro-Optics 3D Printed via Multi-Photon Laser Lithography. Adv. Opt. Mater..

[40] Mainik P., Spiegel C.A., Blasco E. (2023). Recent Advances in Multi-Photon 3D Laser Printing: Active Materials and Applications. Adv. Mater..

[41] Somers P., Münchinger A., Maruo S., Maruo C., Xu X., Wegener M. (2023). The physics of 3D printing with light. Nat. Rev. Phys..

[42] Gissibl T., Thiele S., Herkommer A., Giessen H. (2016). Two-photon direct laser writing of ultracompact multi-lens objectives. Nat. Photonics.

[43] Bauer J., Crook C., Baldacchini T. (2023). A sinterless, low-temperature route to 3D print nanoscale optical-grade glass. Science.

[44] Laakso M., Huang P.H., Edinger P., Hartwig O., Duesberg G.S., Errando-Herranz C., Stemme G., Gylfason K.B., Niklaus F. (2020). Three-dimensional printing of silica-glass structures with submicrometric features. arXiv.

[45] Huang P.H., Laakso M., Edinger P., Hartwig O., Duesberg G., Lai L.L., Mayer J., Nyman J., Errando-Herranz C., Stemme G. (2023). Three-dimensional printing of silica glass with sub-micrometer resolution. Nat. Commun..

[46] Jin F., Liu J., Zhao Y.Y., Dong X.Z., Zheng M.L., Duan X.M. (2022). *λ*/30 inorganic features achieved by multi-photon 3D lithography. Nat. Commun..

[47] Liu T., Tao P., Wang X., Wang H., He M., Wang Q., Cui H., Wang J., Tang Y., Tang J. (2023). Ultrahigh-printing-speed photoresists for additive manufacturing. Nat. Nanotechnol..

[48] Kato J.i., Takeyasu N., Adachi Y., Sun H.B., Kawata S. (2005). Multiple-spot parallel processing for laser micronanofabrication. Appl. Phys. Lett..

[49] Kiefer P., Hahn V., Kalt S., Sun Q., Eggeler Y., Wegener M. (2024). A multi-photon-focus 3D laser printer based on a 3D-printed diffractive optical element and a 3D-printed multi-lens array. Light. Adv. Manuf..

[50] Malinauskas M., Žukauskas A., Bičkauskaitė G., Gadonas R., Juodkazis S. (2010). Mechanisms of three-dimensional structuring of photo-polymers by tightly focussed femtosecond laser pulses. Opt. Express.

[51] Katsura T. (1993). Thermal diffusivity of silica glass at pressures up to 9 GPa. Phys. Chem. Miner..

[52] Uemukai M., Matsumoto N., Suhara T., Nishihara H., Eriksson N., Larsson A. (1998). Monolithically integrated InGaAs-AlGaAs master oscillator power amplifier with grating outcoupler. IEEE Photonics Technol. Lett..

[53] Uenishi K., Uemukai M., Suhara T. (2012). Rotation-Symmetric Multispot Focusing Phase-Shifted Grating Coupler for Integrated Semiconductor Laser. Jpn. J. Appl. Phys..

[54] Gourley K., Golub I., Chebbi B. (2011). Demonstration of a Fresnel axicon. Appl. Opt..

[55] Ovsianikov A., Viertl J., Chichkov B., Oubaha M., MacCraith B., Sakellari I., Giakoumaki A., Gray D., Vamvakaki M., Farsari M. (2008). Ultra-Low Shrinkage Hybrid Photosensitive Material for Two-Photon Polymerization Microfabrication. ACS Nano.

[56] Chatzinikolaidou M., Rekstyte S., Danilevicius P., Pontikoglou C., Papadaki H., Farsari M., Vamvakaki M. (2015). Adhesion and growth of human bone marrow mesenchymal stem cells on precise-geometry 3D organic—Inorganic composite scaffolds for bone repair. Mater. Sci. Eng. C.

[57] Maciulaitis J., Rekstyte S., Bratchikov M., Gudas R., Malinauskas M., Pockevicius A., Usas A., Rimkunas A., Jankauskaite V., Grigaliunas V. (2019). Full length article Customization of direct laser lithography-based 3D scaffolds for optimized in vivo outcome. Appl. Surf. Sci..

[58] Amato L., Gu Y., Bellini N., Eaton S., Cerullo G., Osellame R. (2012). Integrated three-dimensional filter separates nanoscale from microscale elements in a microfluidic chip. Lab Chip.

[59] Zukauskas A., Matulaitiene I., Paipulas D., Niaura G., Malinauskas M., Gadonas R. (2015). Tuning the refractive index in 3D direct laser writing lithography: Towards GRIN microoptics. Laser Photon. Rev..

[60] Skarmoutsou A., Lolas G., Charitidis C., Chatzinikolaidou M., Vamvakaki M., Farsari M. (2013). Nanomechanical properties of hybrid coatings for bone tissue engineering. J. Mechan. Behav. Biomed. Mater..

[61] Gailevicius D., Padolskyte V., Mikoliunaite L., Sakirzanovas S., Juodkazis S., Malinauskas M. (2019). Additive-Manufacturing of 3D Glass-Ceramics down to Nanoscale Resolution. Nanoscale Horiz..

[62] Jonusauskas L., Gailevicius D., Mikoliunaite L., Sakalauskas D., Sakirzanovas S., Juodkazis S., Malinauskas M. (2017). Optically Clear and Resilient Free-Form µ-Optics 3D-Printed via Ultrafast Laser Lithography. Materials.

[63] Sakellari I., Kabouraki E., Karanikolopoulos D., Droulias S., Farsari M., Loukakos P., Vamvakaki M., Gray D. (2019). Quantum dot based 3D printed woodpile photonic crystals tuned for the visible. Nanoscale Adv..

[64] Giakoumaki A., Kenanakis G., Klini A., Androulidaki M., Viskadourakis Z., Farsari M., Selimis A. (2017). 3D micro-structured arrays of Zn0 nanorods. Sci. Rep..

[65] Seniutinas G., Weber A., Padeste C., Sakellari I., Farsari M., David C. (2018). Beyond 100 nm resolution in 3D laser lithography—Post processing solutions. Microelectron. Eng..

[66] Sänger J.C., Pauw B.R., Sturm H., Günster J. (2020). First time additively manufactured advanced ceramics by using two-photon polymerization for powder processing. Open Ceramics.

[67] Gonzalez-Hernandez D., Sanchez-Padilla B., Gailevičius D., Thodika S.C., Juodkazis S., Brasselet E., Malinauskas M. (2023). Single-Step 3D Printing of Micro-Optics with Adjustable Refractive Index by Ultrafast Laser Nanolithography. Adv. Opt. Mater..

[68] Luther-Davies B., Rode A., Madsen N., Gamaly E. (2005). Picosecond high-repetition-rate pulsed laser ablation of dielectrics: The effect of energy accumulation between pulses. Opt. Eng..

[69] Rekštytė S., Jonavicius T., Gailevičius D., Malinauskas M., Mizeikis V., Gamaly E.G., Juodkazis S. (2016). Nanoscale precision of 3D polymerisation via polarisation control. Adv. Opt. Mat..

[70] Ayub S., Gentet L., Fiáth R., Schwaerzle M., Borel M., David F., Barthó P., Ulbert I., Paul O., Ruther P. (2017). Hybrid intracerebral probe with integrated bare LED chips for optogenetic studies. Biomed. Microdev..

[71] Keppeler D., Schwaerzle M., Harczos T., Jablonski L., Dieter A., Wolf B., Ayub S., Vogl C., Wrobel C., Hoch G. (2020). Multichannel optogenetic stimulation of the auditory pathway using microfabricated LED cochlear implants in rodents. Sci. Transl. Med..

[72] Anand V., Maksimovic J., Katkus T., Ng S.H., Ulčinas O., Mikutis M., Baltrukonis J., Urbas A., Šlekys G., Ogura H. (2021). All femtosecond optical pump and x-ray probe: Holey-axicon for free electron lasers. J. Phys. Photonics.

[73] Torcal-Milla F., Lobera J., Lopez A., Palero V., Andres N., Arroyo M. (2022). Mach-Zehnder-based measurement of light emitting diodes temporal coherence. Optik.

[74] Wei S., Cao G., Lin H., Mu H., Liu W., Yuan X., Somekh M., Jia B. (2021). High tolerance detour-phase graphene-oxide flat lens. Photonics Res..

[75] Dharmavarapu R., Bhattacharya S., Juodkazis S. (2018). Diffractive optics for axial intensity shaping of Bessel beams. J. Opt..

[76] Milster T.D., Kim Y.S., Wang Z., Purvin K. (2020). Multiple-order diffractive engineered surface lenses. Appl. Opt..

[77] Apai D., Milster T.D., Kim D.W., Bixel A., Schneider G., Liang R., Arenberg J. (2019). A Thousand Earths: A Very Large Aperture, Ultralight Space Telescope Array for Atmospheric Biosignature Surveys. Astron. J..

[78] Milster T.D., Wang Z., Kim Y.S. (2021). Design aspects of large-aperture MODE lenses. OSA Contin..

[79] Yoshida M., Katsuno S., Inoue T., Gelleta J., Izumi K., De Zoysa M., Ishizaki K., Noda S. (2023). High-brightness scalable continuous-wave single-mode photonic-crystal laser. Nature.

[80] Samsonas D., Skliutas E., Ciburys A., Kontenis L., Gailevičius D., Berzinš J., Narbutis D., Jukna V., Vengris M., Juodkazis S. (2023). 3D nanopolymerization and damage threshold dependence on laser wavelength and pulse duration. Nanophotonics.

[81] Li Z.Z., Fan H., Wang L., Zhang X., Zhao X.J., Yu Y.H., Xu Y.S., Wang Y., Wang X.J., Juodkazis S. (2023). Super stealth dicing of transparent solids with nanometric precision. arXiv.

[82] Gailevičius D., Ryu M., Honda R., Lundgaard S., Suzuki T., Maksimovic J., Hu J., Linklater D.P., Ivanova E.P., Katkus T. (2020). Tilted black-Si: ∼0.45 form-birefringence from sub-wavelength needles. Opt. Express.

[83] Richardson R., Ibbotson M., Thompson A., Wise A., Fallon J. (2020). Optical stimulation of neural tissue. Healthc. Technol. Lett..

[84] Shannon R. (1983). Multichannel electrical stimulation of the auditory nerve in man. II. Channel interaction. Hear. Res..

[85] White M.W., Merzenich M.M., Gardi J.N. (1984). Multichannel cochlear implants. Channel interactions and processor design. Arch. Otolaryngol..

[86] Friesen L., Shannon R., Baskent D., Wang X. (2001). Speech recognition in noise as a function of the number of spectral channels: Comparison of acoustic hearing and cochlear implants. J. Acoust. Soc. Am..

[87] Wilson B., Finley C., Lawson D., Wolford R., Eddington D., Rabinowitz W. (1991). Better speech recognition with cochlear implants. Nature.

[88] Berenstein C., Mens L., Mulder J., Vanpoucke F. (2008). Current steering and current focusing in cochlear implants: Comparison of monopolar, tripolar, and virtual channel electrode configurations. Ear Hear..

[89] Bierer J., Litvak L. (2016). Reducing channel interaction through cochlear implant programming may improve speech perception: Current focusing and channel deactivation. Trends Hear..

[90] Dieter A., Duque-Afonso C., Rankovic V., Jeschke M., Moser T. (2019). Near physiological spectral selectivity of cochlear optogenetics. Nat. Commun..

[91] Dieter A., Klein E., Keppeler D., Jablonski L., Harczos T., Hoch G., Rankovic V., Paul O., Jeschke M., Ruther P. (2020). μ-LED-based optical cochlear implants for spectrally selective activation of the auditory nerve. EMBO Mol. Med..

[92] Azees A., Thompson A., Thomas R., Zhou J., Ruther P., Wise A., Ajay E., Garrett D., Quigley A., Fallon J. (2023). Spread of activation and interaction between channels with multichannel optogenetic stimulation in the mouse cochlea. Hear. Res..

[93] Keppeler D., Kampshoff C., Thirumalai A., Duque-Afonso C., Schaeper J., Quilitz T., Topperwien M., Vogl C., Hessler R., Meyer A. (2021). Multiscale photonic imaging of the native and implanted cochlea. Proc. Natl. Acad. Sci. USA.

[94] Jurgens T., Hohmann V., Buchner A., Nogueira W. (2018). The effects of electrical field spatial spread and some cognitive factors on speech-in-noise performance of individual cochlear implant users-A computer model study. PLoS ONE.

[95] Khurana L., Jablonski D.K.L., Moser T. (2022). Model-based prediction of optogenetic sound encoding in the human cochlea by future optical cochlear implants. Comput. Struct. Biotechnol. J..

[96] Dharmavarapu R., Ng S.H., Eftekhari F., Juodkazis S., Bhattacharya S. (2020). MetaOptics: Opensource software for designing metasurface optical element GDSII layouts. Opt. Express.

[97] Dharmavarapu R., Izumi K., Katayama I., Ng S.H., Vongsvivut J., Tobin M.J., Kuchmizhak A., Nishijima Y., Bhattacharya S., Juodkazis S. (2019). Dielectric cross-shaped-resonator-based metasurface for vortex beam generation at mid-IR and THz wavelengths. Nanophotonics.

